# Expression and localization of DAB1 and Reelin during normal human kidney development

**DOI:** 10.3325/cmj.2019.60.521

**Published:** 2019-12

**Authors:** Anita Racetin, Marija Jurić, Natalija Filipović, Ivana Šolić, Ivona Kosović, Merica Glavina Durdov, Nenad Kunac, Sandra Zekić Tomaš, Marijan Saraga, Violeta Šoljić, Vlatka Martinović, Joško Petričević, Ivana Restović, Valentina Lasić, Sandra Kostić, Boris Kablar, Koichiro Watanabe, Yu Katsuyama, Mirna Saraga Babić, Katarina Vukojević

**Affiliations:** 1 Department of Anatomy, Histology and Embryology, University of Split School of Medicine, Split, Croatia; 2 Department of Pathology, Clinical Hospital Center Split, Split, Croatia; 3 Department of Pediatrics, Clinical Hospital Center Split, Split, Croatia; 4 University of Mostar, School of Medicine, University Hospital Center Mostar, Mostar, Bosnia and Herzegovina; 5 Faculty of Humanities and Social Sciences, Split, Croatia; 6 Departments of Neuroscience/Anatomy and Pathology, Dalhousie University, Halifax, Canada; 7 Department of Anatomy, Shiga University of Medical Science, Otsu, Japan

## Abstract

**Aim:**

To explore the spatial and temporal expression patterns of DAB1 and Reelin in the developing and postnatal healthy human kidneys as potential determinants of kidney development.

**Methods:**

Paraffin-embedded fetal kidney tissue between the 13/14th and 38th developmental weeks (dw) and postnatal tissue at 1.5 and 7 years were stained with DAB1 and Reelin antibodies by double immunofluorescence.

**Results:**

During the fetal kidney development and postnatal period, DAB1 and Reelin showed specific spatial expression pattern and diverse fluorescence intensity. During the fetal period, DAB1 was strongly expressed in the distal convoluted tubules (DCT), with strong reactivity, and diversely in the proximal convoluted tubules (PCT) and glomeruli. In the postnatal period, DAB1 expression decreased. The strongest Reelin expression in early fetal stages was observed in the PCT. In the postnatal period, Reelin expression decreased dramatically in all observed structures. These two markers were colocalized during early developmental stages, mostly in PCT, DCT, and podocytes.

**Conclusion:**

The appearance of DAB1 and Reelin during fetal kidney development confirms their potential significant role in the formation of kidney structure or function. High DAB1 expression in the DCT implies its regulatory role in tubular formation or function maintenance during development. Reelin was highly expressed in human kidneys at early fetal stages, mostly in the PCT, while at later fetal stages and postnatal period its expression decreased.

The human kidneys develop from the intermediate mesoderm (1) at about the third week of gestation (gw), and nephrogenesis finishes before birth (2), around the 36th week of gestation (3). At birth, a full-term newborn has a definite number of nephrons in each kidney, with no further increase in their number, only in size and functional maturation (2). The permanent kidney, metanephros, becomes functional at around the 10th gw (4), when urine production starts (2). The metanephros consists of nephrons and collecting ducts, which have different developmental origin.

Canonical Reelin/ApoER2 or VLDLR/DAB1 pathway may trigger distinct signaling cascades, which regulate specific biologic activities at different times during embryonic development. Reelin is a large extracellular glycoprotein that binds to apolipoprotein E receptor 2 (Apo-ER2) or to very-low-density lipoprotein receptor (VLDLR) (5). Reelin interactions lead to receptor dimerization and tyrosine phosphorylation of the downstream cytoplasmic adaptor protein DAB1 (6-8) by SRC-family kinases (SFKs), FYN (proto-oncogene tyrosine-protein kinase) and SRC (non-receptor tyrosine protein kinase) (7). The inactivation of *Dab1* in *scrambler* or *yotari* mouse generates a phenotype similar to that of Reelin-deficient mice (9,10). Beside the neural tissues, the presence of DAB1 is confirmed in human breast cancer (11), mouse podocytes (12), and rodent intestine (13).

Mutations in *RELN*, a gene coding for Reelin, result in a specific lissencephaly, with mental retardation and severe abnormalities of the cerebellum, hippocampus, and brain stem (14). The presence of Reelin has also been confirmed in other developing mouse tissues, such as the optic and olfactory nerve fibers, spinal cord, limb bones, temporal bones, liver, spleen (15), as well as in adult mammalian blood, liver, pituitary, and adrenal glands (16). Additionally, the localization of *Reelin* mRNA was confirmed at E14.5-16.5 of mouse kidney development (corresponding to the human 7th-8th week) (15). Reelin was found to be expressed in the interstitial region and coelomic mesothelium, but not in the ureteric bud, metanephric blastema, or nephrons. In the adult mouse kidney, Reelin was expressed by some endothelial cells along blood vessels (15).

It is well known that DAB1 and Reelin have pivotal roles during brain development, both in mice and humans, particularly in the organization of the brain architecture patterns (17). Interestingly, DAB1 and Reelin are expressed in tissues other than brain, thus more systematic data on their extraneural localization during development are welcome. Intriguing evidence on potential functional interplay of DAB1 and Reelin in mouse podocytes (12) raises the possibility that these two proteins have more important roles in mammalian kidney than anticipated. We assume that DAB1 and Reelin may have an important role during kidney development. Therefore, the aim of this study was to analyze the expression, localization, and possible colocalization of DAB1 and Reelin in fetal stages of kidney development following the beginning of urine production and in postnatal stages of the human kidney development. 

## Materials and methods

### Human materials

Kidney samples of fetuses aged between the 13/14th and 38th dw obtained after spontaneous abortions and kidney tissues of children aged 1.5 and 7 years obtained after accidental death were collected from the Department of Pathology, University Hospital Center Split. The fetuses were collected and examined macroscopically and measured to exclude samples with abnormalities between 1998 and 2006. Only normal fetuses, without any sign of abnormality and macerations, were used (18). All fetal and postnatal tissues were treated as postmortem material with the permission of the Ethics Committee of University Hospital Center Split (class: 003-08/16-03/0001, approval number: 2181-198-03-04-16-0024), in accordance with the 1964 Helsinki Declaration (19). The postovulatory age was estimated by the menstrual cycle data and correlated with fetal biparietal diameter values (20) (Table 1).

**Table 1:** The human conceptuses analyzed in the study

Developmental week	Number of fetuses	Gestation week	Biparietal diameter (mm)	Femur length (mm)
13-14	3	15-16	34-37	19-22
15	3	17	40	24
16	3	18	43	27
21-22	3	23-24	59-62	41-43
38	3	40	97	73

### Immunohistochemical staining

Kidney tissues were dissected and fixed in 4% paraformaldehyde in phosphate buffer and dehydrated in 100% ethanol. They were embedded in paraffin wax, serially sectioned at 5 μm, and mounted on glass slides. The sections were deparaffinized in xylene and rehydrated in ethanol and water as we described previously (21-23). After washing in phosphate-buffered saline (PBS), the sections were incubated in humid chamber over night with primary antibodies: Mouse Monoclonal Reelin antibody (1:70 dilutions; sc-25346, Santa Cruz Biotechnology, Dallas, TX, USA), Rabbit Polyclonal Anti-DAB1 (phospho Y232) antibody (1:400 dilutions; ab 78200, Abcam, Cambridge, UK). After washing in PBS, secondary antibodies, Donkey Anti-Rabbit IgG H&L, Alexa Fluor 488 (dilution 1:400, ab 150073, Abcam) and Goat Anti-Mouse IgG H&L, TRITC (dilution 1:400, ab 6786, Abcam) were applied for one hour. The nuclei were stained with DAPI for 2 minutes, washed in PBS, and coverslipped. Controls for specificity of staining, with omission of the primary antibody, were included in the staining procedure to exclude nonspecific staining.

### Data acquisition and semi-quantification analysis

We used three samples per group and 10 sections per each sample. The analysis was performed with the fluorescence microscope (Olympus BX61, Tokyo, Japan) equipped with a DP71 digital camera (Olympus). Images were analyzed with ImageJ Software and Adobe Photoshop (Adobe, San Jose, CA, USA). The staining intensity of kidney structures was semiquantitatively organized into four groups: the absence of any reactivity (−), mild reactivity (+), moderate reactivity (++), and strong reactivity (+++) (Table 2). The percentage of DAB1 and Reelin immunoreactive cells was calculated. For each investigated period, we captured at least twenty images per different kidney structure: proximal convoluted tubules (PCT), distal convoluted tubules (DCT), and glomeruli at 40 × objective magnification. We averaged the number of positive cells in each kidney structure from 10 sections. Any level of cytoplasmic or membrane staining with the used markers was regarded as positive. Two investigators analyzed the images independently.

**Table 2:** Staining intensity to specific antibodies in the human kidney development during the 13/14th to 38th development week and 1.5 and 7 years of postnatal development. Twenty kidney structures are analyzed (20 PCT, 20 DCT, 20 G) in each sample*^†^

	Reelin							DAB1						
Structure	13/14 w	15 w	16 w	21/22 w	38 w	1.5 y	7 y	13/14 w	15 w	16w	21/22w	38w	1.5 y	7 y
G	+	+	+	+	+	+	+	+	+/++	+	+/++	++/+++	**+**	**++**
PCT	++/ +++	++	++	+/++	++	++	+/++	++/+++	+/++	++/ +++	++/+++	+	**+/++**	**+**
DCT	++/ +++	+++	+/++	+/++	+	-	++	++/ +++	+++	+++	+++	+++	**++/ +++**	**++/ +++**

*G – glomeruli, PCT – proximal convoluted tubules, DCT – distal convoluted tubules, w – week of development, y – years of postnatal development.

†Three pluses indicate strong reactivity; two pluses indicate moderate reactivity; one plus indicates mild reactivity; minus indicates no reactivity.

### Statistical analyses

Kruskal-Wallis test followed by Dunn’s multiple comparison test was used to examine the differences in the percentage of positive cells between PCT, DCT, and glomeruli at all time points. The percentage of positive cells was expressed as median ± interquartile range. The level of significance was set at *P* < 0.05. The analysis was conducted in GraphPad Software (GraphPad Software, La Jolla, CA, USA).

## Results

The localization and semi-quantification of DAB1 and Reelin expression were analyzed in the already formed nephrons with the clearly differentiated glomeruli, PCT, and DCT. In the fetal period of kidney development, diverse stages of nephrogenesis were identified, including ureteric bud ingrowth, metanephric cap condensation, renal vesicles formation, and comma- and S-shaped glomeruli, but these structures were not included in the current analysis.

### 13th-14th developmental week

**Figure 1:**
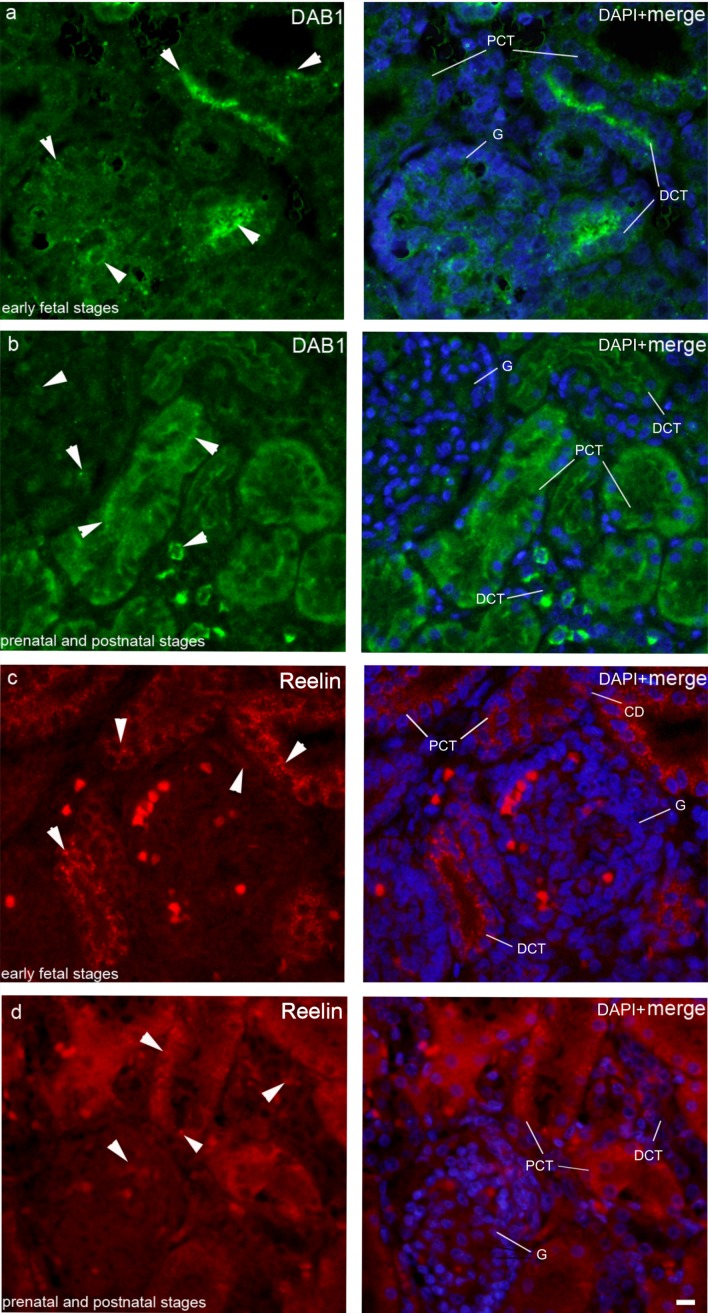
Immunofluorescence staining of differentiating and mature human kidneys with DAB1 and Reelin antibodies. Nuclear DNA DAPI staining merged with DAB1 and Reelin immunofluorescence is shown in parallel (merge). Observed time points were divided into two periods, early fetal period (**A**,**C**) and prenatal and postnatal period (**B**,**D**). Samples at the 13th-14th dw, 15th dw, 16th dw, and 21th-22nd dw represent early fetal period, while samples at the 38th dw, 1.5 year, and 7 year represent prenatal and postnatal period. During the early fetal period, DAB1 was strongly expressed on the apical membranes of the distal convoluted tubules (DCT) and moderately expressed in the cytoplasm of the convoluted tubules (PCT) and glomeruli, mostly in visceral podocytes (arrowheads). During the prenatal and postnatal period, DAB1 was strongly expressed on the apical membranes of the PCT, while in the visceral podocytes and in the DCT it was moderately expressed on the whole cell membrane with high reactivity (arrowheads). During the early fetal period, Reelin was strongly expressed throughout the cytoplasm of the DCT, PCT, and collecting ducts, while it was mildly expressed in the glomeruli, mostly in the parietal layer of the Bowman capsule (arrowheads). In the prenatal and postnatal period, Reelin expression decreased. It was mildly expressed in the cytoplasm of the PCT, DCT, and visceral layer of the Bowman capsule. Scale bar is 20 μm, refers to all images.

**Figure 2:**
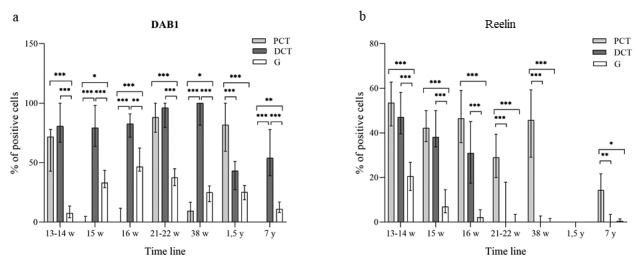
The percentage of DAB1 (**A**) and Reelin (**B**) positive cells in the proximal convoluted tubules (PCT), distal convoluted tubules (DCT), and glomeruli (**G**) of developing human kidneys between the 13th and 38th developmental week, and in postnatal healthy kidneys at the age of 1.5 and 7 years. Data are presented as median ± interquartile range (vertical line). Significant differences between PCT, DCT, and G at the same time point are indicated by *P < 0.01, **P < 0.001, ***P < 0.0001 (Kruskal-Wallis test followed by Dunn’s multiple comparison test). At each time point 20 PCT, DCT, and glomeruli were assessed.

In the 13th and 14th dw, cortical parts of human kidneys contained collecting ducts (originating from branching ureteric bud) and nephrons in different stages of development, including renal vesicle, S-form, and C-forms of nephrons, as well as immature glomeruli. More mature forms of glomeruli and nephron tubules in the advanced stage of differentiation were observed closer to the developing medulla. DAB1 was expressed mostly on the apical and lateral parts of cell membranes, or within the cytoplasm of the DCT (Figure 1A). The percentage of positive cells was 81.89% (Figure 2A), with moderate to high reactivity (Table 2). Similar patterns of distribution and reactivity were observed also in the PCT, but with a lower percentage of positive cells (62.04%) (Figure 2A). In the glomeruli, the percentage of positive cells and reactivity (Table 2) were lowest (8.33%) (Figure 2A). The percentage of positive cells significantly differed between the PCT and glomeruli and between DCT and glomeruli (*P* < 0.0001, Figure 2A).

**Figure 3:**
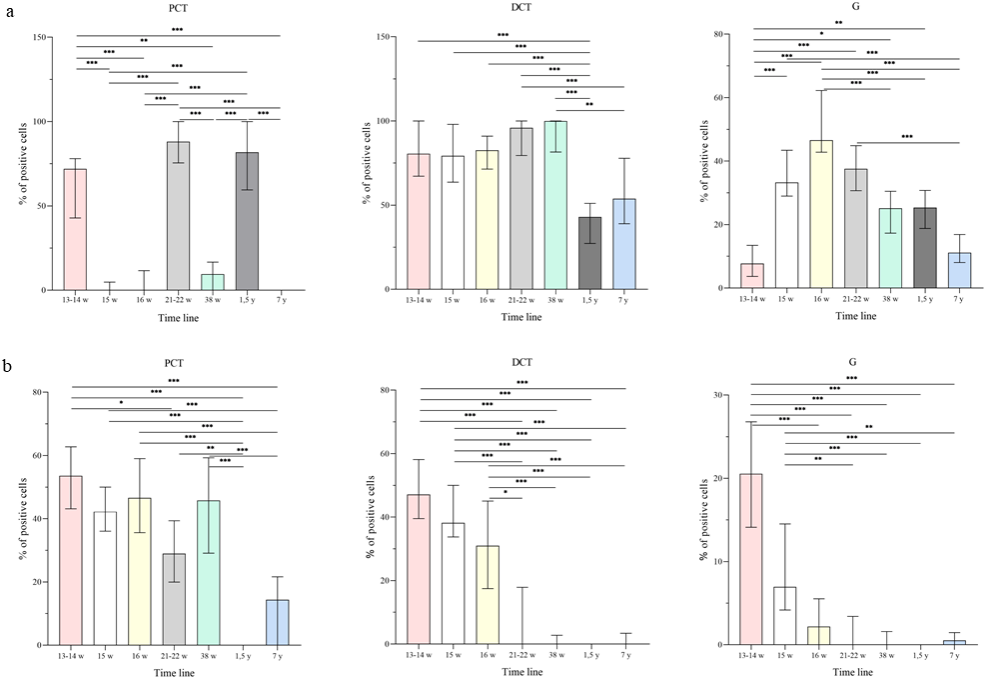
The distribution of percentage of DAB1 (**A**) and Reelin (**B**) positive cells in the proximal convoluted tubules (PCT), distal convoluted tubules (DCT), and glomeruli (G) in developing human kidneys between the 13th-14th and 38th developmental week, and in postnatal healthy kidneys at the age of 1.5 and 7 years. Data are presented as the median ± interquartile range (vertical line). Significant differences between the PCT, DCT, and G at different time points are indicated by *P < 0.01, **P < 0.001, ***P < 0.0001 (Kruskal-Wallis test followed by Dunn’s multiple comparison test). At each time point 20 PCT, DCT and glomeruli were assessed.

In this period, Reelin expression was highest during fetal development in all observed structures (Kruskal-Wallis, Figure 3B). The percentage of positive cells in both PCT and DCT was 50% (Figure 2B), and the reactivity was moderate to high (Table 2) and scattered in the cytoplasm (Figure 1C). In the glomeruli, the percentage of positive cells was lowest (19.30%) (Figure 2B), with mild reactivity (Table 2). The percentage of positive cells significantly differed between the PCT and glomeruli and between DCT and glomeruli (*P* < 0.0001, Figure 2B). DAB1 and Reelin were colocalized in the PCT and DCT (not shown).

### 15th and 16th developmental week

The maturity of kidney tissue in the 15th to 16th dw did not change significantly when compared with the earlier developmental stage. These two developmental weeks were observed together because there were no significant differences in the percentage of immunoreactive cells (Figure 3AB), their spatial distribution, or staining intensity between DAB1 and Reelin (Table 2). In the DCT, there was a high percentage (71.61%-80.85%) of DAB1 positive cells (Figure 2A), with strong reactivity at apical parts of cell membranes (Figure 1A). In the PCT, DAB1 was expressed in the apical cell membrane and cytoplasm (Figure 1A), with mild to moderate reactivity (Table 2). In the glomeruli, it was present mostly in visceral podocytes (Figure 1A), with mild to moderate reactivity (Table 2). The percentage of positive cells significantly differed between the PCT and DCT (*P* < 0.0001, Figure 2A), between the PCT and glomeruli (*P* < 0.01, Figure 2A), and between the DCT and glomeruli (*P* < 0.0001, Figure 2A). In comparison with 13th-14th dw, the percentage and intensity of staining significantly decreased in the PCT (*P* < 0.0001, Figure 3A) and significantly increased in the glomeruli (*P* < 0.0001, Figure 3A).

**Figure 4:**
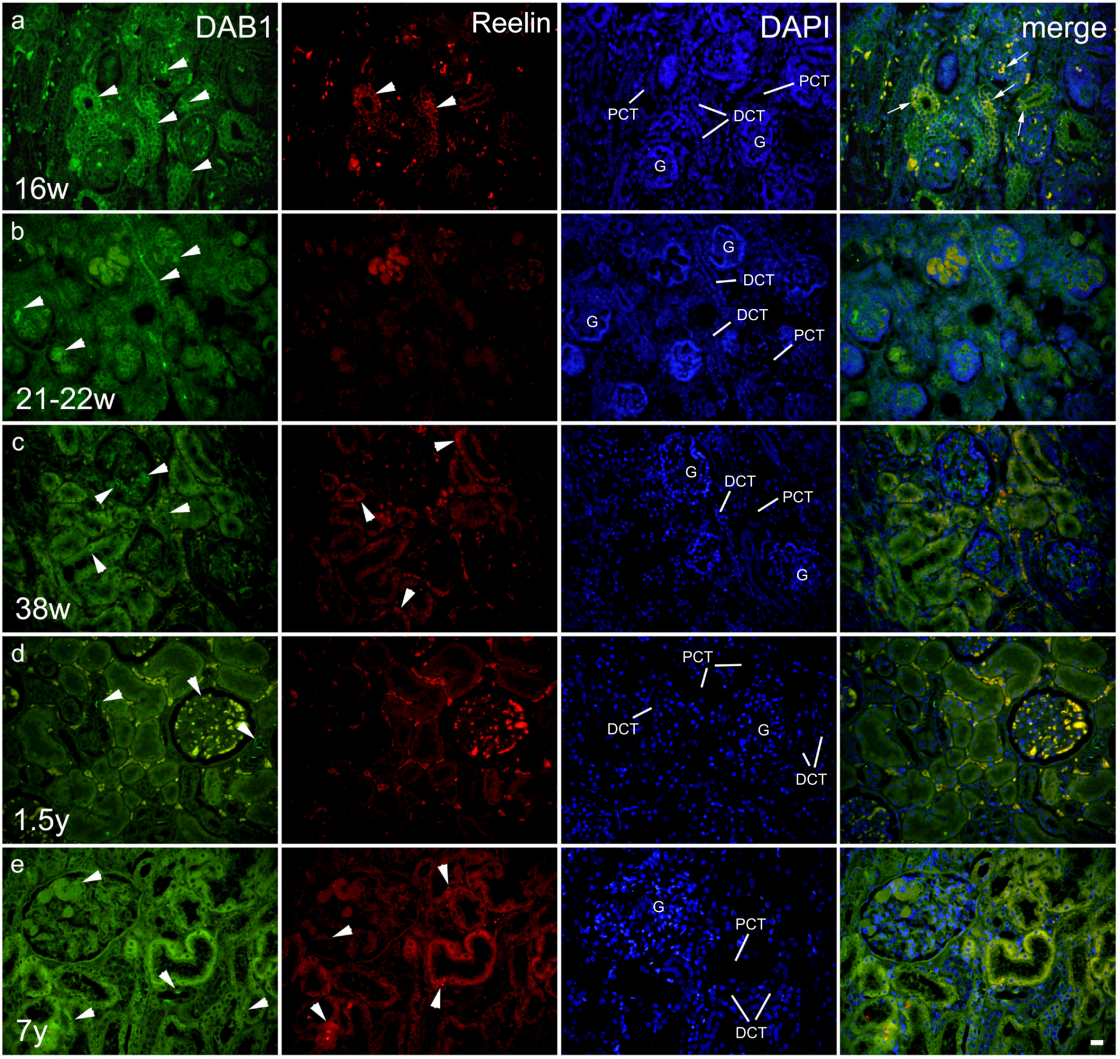
Double immunofluorescence of differentiating and mature human kidneys with DAB1 and Reelin antibodies. Nuclear DNA DAPI staining merged with DAB1 and Reelin immunofluorescence is shown in parallel (merge). (**A**) In the 16th developmental week (dw), DAB1 was strongly expressed (arrowhead) on the membranes of the distal convoluted tubules (DCT) and glomeruli (G) and weakly on the membranes of the proximal convoluted tubules (PCT). Reelin was moderately expressed (arrowhead) on the membranes of the PCT and DCT. DAPI nuclear staining revealed the colocalization of DAB1 and Reelin in the PCT, DCT, and visceral podocytes (arrows). (**B**) In the 21th-22nd dw, DAB1 (arrowhead) was strongly expressed on the membranes of the PCT, DCT, and in G. Reelin was not expressed in these structures. There was no colocalization of DAB1 and Reelin. (**C**) In the 38th developmental week, DAB1 was strongly expressed (arrowhead) on the membranes of the DCT and in visceral podocytes, while it was mildly expressed in the cytoplasm of PCT. Reelin (arrowhead) was weakly expressed in the cytoplasm of the PCT. DAPI nuclear staining revealed poor colocalization of DAB1 and Reelin in the PCT (arrows). (**D**) At the age of 1.5 year, DAB1 was strongly expressed (arrows) on the membranes of the DCT and in podocytes in the G. Reelin was not expressed in these structures. DAPI nuclear staining revealed no colocalization of DAB1 and Reelin. (**E**) At the age of 7 years, DAB1 was strongly expressed (arrows) on the membranes of the DCT and in podocytes in the G. Reelin was weakly expressed in the cytoplasm of the PCT. DAPI nuclear staining revealed no colocalization of DAB1 and Reelin. Scale bar is 20 μm, refers to all images.

Reelin showed a similar spatial distribution and percentage of positive cells (Figure 2B). The percentage of positive cells in the PCT was 35%-40% (Figure 2B). The reactivity was mild to moderate (Table 2) and the staining was mostly spread through the cytoplasm (Figure 1C). In the DCT and collecting ducts, a slightly lower percentage was observed (Figure 2B), around 30%. The reactivity of staining was mostly moderate (Table 2), and the staining was diffusely distributed in the cytoplasm (Figure 1C). In the glomeruli, the percentage was lower than 10% (Figure 2B), with mild reactivity (Table 2). The percentage of positive cells was significantly different between the PCT and glomeruli, and between the DCT and glomeruli (*P* < 0.0001, Figure 2B) during both dws. DAB1 and Reelin were colocalized in the PCT and DCT (Figure 4A).

### 21st-22nd developmental week

In the 21st to 22nd dw, the nephrogenic zone was still present in the cortical part of the kidney, containing nephrons in different stages of development. However, much more mature forms of nephrons were located close to the medulla. DAB1 was highly expressed (86.8%) in the DCT (Figure 2A), with moderate to high reactivity (Table 2). Staining was usually concentrated at the apical and lateral parts of cell membranes, and less in the cytoplasm (Figure 1A). Almost the same distribution and reactivity (Figure 1A), with a high percentage (84.79%) of positive cells, were observed in the PCT (Figure 2A). The percentage of positive cells in the PCT in these developmental weeks was significantly higher in comparison with 15th-16th dw (*P* < 0.0001, Figure 3A). Also, compared with 15th and 16th dw, the percentage of positive cells non-significantly decreased in the glomeruli, mostly in visceral podocytes (*P* > 0.05, Figure 3A). Distribution and intensity patterns were similar as in previously described developmental stages. The percentage of positive cells significantly differed between the PCT and glomeruli and between the DCT and glomeruli (*P* < 0.0001, Figure 2A).

Reelin was poorly expressed, with mild reactivity (Table 2) in the DCT and glomeruli, while the percentage of positive cells in the PCT was nearly 30% (Figure 2B). Staining was localized within the cytoplasm (Figure 1C) with mild to moderate reactivity (Table 2). The percentage of positive cells significantly differed between the PCT and DCT, and PCT and glomeruli (*P* < 0.0001, Figure 2B). The percentage of positive cells in the DCT in these developmental weeks was significantly lower in comparison with 15th-16th dw (*P* < 0.0001, Figure 3B). DAB1 and Reelin were colocalized only occasionally.

### 38th developmental week

In the 38th dw, there was no nephrogenic zone in the cortical part of the kidney. The nephrons displayed morphological characteristics of maturity. DAB1 was highly expressed in the DCT (Figure 2A), with strong reactivity (Table 2), mostly at apical cell membranes (Figure 1B). The percentage of DAB1 positive cells in the PCT was significantly lower compared with the 21th-22nd dw (*P* < 0.0001, Figure 3A). Only a small amount (10.45%) of mildly positive staining was observed (Figure 4C). In the glomeruli, the percentage of positive cells was about 20%, with moderate reactivity (Figure 1B). The percentage of positive cells significantly differed between the PCT and DCT, between the DCT and glomeruli (*P* < 0.0001, Figure 2A), and between the PCT and glomeruli (*P* < 0.01, Figure 2A).

The percentage of Reelin positive cells in the PCT was about 40% (Figure 2B), with moderate reactivity (Table 2), while in the DCT and glomeruli it was negligible (Figure 4C), with mild reactivity (Table 2). In all kidney structures, Reelin expression was spread throughout the cytoplasm (Figure 1D). The percentage of positive cells significantly differed between the PCT and DCT, and between the PCT and glomeruli (*P* < 0.0001, Figure 2B). DAB1 and Reelin were rarely colocalized (Figure 4C).

### 1.5 years

In the postnatal period, nephrogenesis was completed, and the mature forms of nephrons characterized the well differentiated cortex and medulla. In comparison with the 21th-22nd dw, the percentage of DAB1 positive cells in the DCT significantly decreased (*P* < 0.0001, Figure 3A), with 36.52% of positive cells. Reactivity was still strong (Table 2), dispersed mostly on the apical cell membrane (Figure 4D). In comparison with the 38th dw, the percentage of positive cells in the PCT significantly increased (*P* < 0.0001, Figure 3A), with 80% of positive cells with moderate reactivity throughout the cytoplasm and on the apical membrane (Figure 1B). In the glomeruli, the percentage of positive cells was the almost same as in the 38th dw, and the staining was mostly concentrated in visceral podocytes (Figure 1B), with mild reactivity (Table 2). The percentage of positive cells significantly differed between the PCT and DCT, and between the PCT and glomeruli (*P* < 0.0001, Figure 2A).

Reelin-positive cells in the PCT and DCT were observed occasionally (Figure 1D). The percentage of Reelin positive cells in the PCT significantly decreased in comparison with the 38th dw (*P* < 0.0001, Figure 3B). DAB1 and Reelin were not colocalized (Figure 4D).

### 7 years

The histological structure of 7-year kidney did not considerably morphologically differ compared with that from the previous postnatal period. DAB1 was moderately expressed (Table 2) in the DCT, with about 60% of positive cells (Figure 2A). The staining was concentrated on the membrane or spread through the cytoplasm (Figure 4d). In the PCT, compared with the previous stage, the percentage of positive cells significantly decreased (*P* < 0.0001, Figure 3A), with 3.6% of positive cells. Reactivity was mild (Table 2) and found only in the cytoplasm (Figure 4E). In the glomeruli, the percentage of positive cells was 10% (Figure 2A), and the staining was mostly concentrated in visceral podocytes (Figure 1B), with mild reactivity (Table 2). The percentage of positive cells significantly differed between the PCT and DCT, between the DCT and glomeruli (*P* < 0.0001, Figure 2A), and between the PCT and glomeruli (*P* < 0.001, Figure 2A).

Reelin was poorly expressed in all structures (Figure 2B). Low percentage of positive cells with mild reactivity (Table 2) was found in the cytoplasm of PCT and DCT, while in the glomeruli only a few isolated signals were observed (Figure 4E). The percentage of positive cells was significantly higher in the PCT than in the DCT and glomeruli (*P* < 0.001, Figure 2B). DAB1 and Reelin were not colocalized (Figure 4E).

## Discussion

Except in mouse podocytes (13), almost no findings have been reported about DAB1’s function or expression in kidney tissue, particularly in human fetal or adult kidneys. Our study showed high DAB1 expression during fetal stages of human kidney development. The highest expression was observed in the DCT, mostly at apical and lateral parts of cell membranes, which contain a large number of ion exchange protein channels, necessary for body fluid and ion homeostasis maintenance. DAB1 might trigger downstream pathways, which influence the expression or function of those transmembrane ion exchange proteins. DAB1 decrease in the DCT of postnatal kidneys might indicate that its function is weaker in adulthood compared with kidney development. In the PCT, DAB1 expression varied, with large fluctuation between the analyzed developmental weeks, which could be associated with specific developmental processes occurring at each observed time point. In our study, DAB1 was largely expressed in kidney tissue during all stages of fetal development, suggesting its possible participation in morphogenesis and establishing of nephron function, while DAB1 mutation might disrupt downstream signaling cascades, leading to disturbed kidney function.

DAB1 is an intracellular adaptor protein that may trigger different downstream pathways, such as CRKs/C3G/Rap1/N-Cadherin, PI3K/Akt/mTOR, and MEK/ERK, necessary for cell response to diverse extracellular stimuli (24). Morphological brain abnormalities in *reeler* (Reelin knockout mice), *yotari* (*Dab1* knockout mice), and *Reelin/Dab1* mutants were identical (25), suggesting that Dab1 and Reelin are on the same linear signaling pathway. So far, DAB1 has been poorly investigated in non-neural tissues. Its presence was confirmed in human breast cancer (11), rodent intestine (13), mammary gland development, cartilage and tendon differentiation, and during odontogenesis (26-29). Previous investigations confirmed that DAB1 triggers Crks adaptor proteins, which first activate C3G and subsequently Rap1 (30,31). Rap1 activates N-cadherin localized in the tubules of healthy adult human kidneys, whereas in acute kidney injury its expression is abolished (32). DAB1 may also trigger MAP kinase pathways (MAPK), such as p38MAPK and ERK, the expression of which was confirmed during rat kidney growth and development, and which seems to play a role in inflammation, cell growth, and development (33). ERK appears to play a role in nephrogenesis, and p38 in kidney growth and nephrogenesis (33). In addition, p38 expression increases in human diabetic nephropathy (34) and human crescentic glomerulonephritis (35), suggesting an important pathogenic role of p38 MAPK. DAB1 also triggers PI3K/Akt/mTOR signaling pathway. This pathway was one of the main regulators of cell proliferation and thus a target for multiple therapeutic strategies (36). Mutations in the PI3K/Akt/mTOR pathway may cause polycystic kidney disease (37). Some experiments revealed that the inhibition of PI3K pathway in kidney organ cultures suggests that primary UB formation depends on chemotactic cell motility by this pathway (38). Similar experiments with MEK inhibitors imply that MAPK pathway is also required for UB morphogenesis (39,40). Namely, the loss of its activity prevents the generation of new branches, while allowing bud elongation (41). Interestingly, Reelin/DAB1 canonical pathway triggers the same downstream factors as GDNF/RET pathway, including MAPK, ERK, and PI3K (42). These factors triggered by GDNF/RET pathway are involved in cell proliferation, migration, degradation, and ureteric epithelial branching (42).

Our study showed that Reelin emerged in nephrons, mostly in the PCT and DCT, with moderate reactivity. Reelin expression has been already confirmed in non-neural developing tissues but not in the ureteric bud, metanephric blastema, or nephrons (15), which is contrary to our findings. This might be explained by differences between mouse and human kidney samples. After double immunofluorescence staining of fetal and postnatal human kidneys with DAB1 and Reelin antibodies, our results showed that in the healthy human kidneys DAB1 and Reelin were colocalized only during early fetal stages. After the 16th dw, colocalization decreased and disappeared almost completely before birth. After the 38th dw, Reelin expression was low, while DAB1 was still expressed in a large amount. These results indicate that DAB1 can be triggered by some other pathway containing Src family kinases. Reelin was largely expressed in early fetal stages, suggesting its participation in nephrogenesis. In the postnatal period, there was almost no Reelin expression, except a small amount in the PCT of the 7-year-old sample. This indicates that Reelin may not be involved in kidney maturation or the maintenance of kidney function in early childhood.

Studying of DAB1-Reelin expression and co-expression during human kidney development requires fetal kidney samples. Acquisition of greater number of these samples poses a significant limitation due to ethical reasons. For this study we managed to acquire three samples per each kidney developmental stage, but the use of more samples could increase the reliability of findings.

In conclusion, DAB1 and Reelin were largely expressed during normal fetal kidney development and less so in postnatal healthy kidneys. As DAB1 was highly expressed during all observed fetal stages, mostly in the DCT, it may have an important role in tubular formation or maintenance of function. Reelin was highly expressed in human kidneys at early fetal stages, mostly in the PCT, while its expression decreased at later fetal stages and postnatal period. Colocalization of DAB1 and Reelin was not confirmed during all dws, implicating that DAB1 can be triggered by some other factors rather than only by Reelin. Further investigations are necessary to find out what other extracellular signaling pathways control intracellular activation of DAB1 and to clarify its influence in kidney development.
